# Behavior of Solvent-Exposed Hydrophobic Groove in the Anti-Apoptotic Bcl-X_L_ Protein: Clues for Its Ability to Bind Diverse BH3 Ligands from MD Simulations

**DOI:** 10.1371/journal.pone.0054397

**Published:** 2013-02-28

**Authors:** Dilraj Lama, Vivek Modi, Ramasubbu Sankararamakrishnan

**Affiliations:** Department of Biological Sciences & Bioengineering, Indian Institute of Technology Kanpur, Kanpur, India; Indian Institute of Science, India

## Abstract

Bcl-X_L_ is a member of Bcl-2 family of proteins involved in the regulation of intrinsic pathway of apoptosis. Its overexpression in many human cancers makes it an important target for anti-cancer drugs. Bcl-X_L_ interacts with the BH3 domain of several pro-apoptotic Bcl-2 partners. This helical bundle protein has a pronounced hydrophobic groove which acts as a binding region for the BH3 domains. Eight independent molecular dynamics simulations of the apo/holo forms of Bcl-X_L_ were carried out to investigate the behavior of solvent-exposed hydrophobic groove. The simulations used either a twin-range cut-off or particle mesh Ewald (PME) scheme to treat long-range interactions. Destabilization of the BH3 domain-containing helix H2 was observed in all four twin-range cut-off simulations. Most of the other major helices remained stable. The unwinding of H2 can be related to the ability of Bcl-X_L_ to bind diverse BH3 ligands. The loss of helical character can also be linked to the formation of homo- or hetero-dimers in Bcl-2 proteins. Several experimental studies have suggested that exposure of BH3 domain is a crucial event before they form dimers. Thus unwinding of H2 seems to be functionally very important. The four PME simulations, however, revealed a stable helix H2. It is possible that the H2 unfolding might occur in PME simulations at longer time scales. Hydrophobic residues in the hydrophobic groove are involved in stable interactions among themselves. The solvent accessible surface areas of bulky hydrophobic residues in the groove are significantly buried by the loop LB connecting the helix H2 and subsequent helix. These observations help to understand how the hydrophobic patch in Bcl-X_L_ remains stable in the solvent-exposed state. We suggest that both the destabilization of helix H2 and the conformational heterogeneity of loop LB are important factors for binding of diverse ligands in the hydrophobic groove of Bcl-X_L_.

## Introduction

The role of Bcl-2 family in regulating the mitochondrial outer membrane permeabilization, an important step in the cell death process, is well established [1], [2], [3], [4], [5]. Among the functionally apposing Bcl-2 members, the anti-apoptotic Bcl-X_L_ protein is one of the first proteins from the Bcl-2 family that has been investigated by several researchers [6], [7], [8], [9], [10]. This protein has been specifically shown to be over-expressed in several human cancers including lung [11], colon [12], pancreatic [13], breast [14] and prostate [15] cancers. Hence, Bcl-X_L_ like other anti-apoptotic Bcl-2 members is an attractive target for anti-cancer drugs [16], [17]. Structural knowledge of this protein has helped to understand how Bcl-X_L_ is recognized by its pro-apoptotic partners which is a crucial step for the cell survival [18]. The pronounced hydrophobic groove present in this helical bundle protein acts as a binding region for the BH3 domain of pro-apoptotic Bcl-2 members. Experimental studies have shown that the BH3 peptides derived from the pro-apoptotic proteins have the ability to elicit the same biological response as that of the parent proteins [19]. As of December 2012, 50 Bcl-X_L_ structures have been deposited in the Protein Data Bank [20] including 30 structures in which Bcl-X_L_ is in complex with a BH3 peptide or an inhibitor molecule. However, biochemical and pharmacological studies have demonstrated that different pro-apoptotic proteins exhibit distinct binding affinities for Bcl-X_L_ although experimentally determined structures show that they all bind to the same hydrophobic groove [19], [21], [22]. Structure-based drug design approach combined with docking simulations has been used to identify and/or design small molecule inhibitors that can target Bcl-X_L_ [23]. Design of molecules mimicking the BH3 domains of pro-apoptotic Bcl-2 proteins [24], [25], [26], pharmacophore-based database searching [27], [28] and fragment-based building of bioactive inhibitors [29] are some of the approaches used in designing and synthesizing Bcl-X_L_-specific inhibitors.

Three factors could be important for the pro-apoptotic proteins to recognize and bind particular anti-apoptotic Bcl-2 proteins with specific binding affinities.

- Specific interactions could help the ligands to bind tightly to the target proteins. Among the different computational approaches that investigated the differential binding affinities, molecular dynamics (MD) simulations have identified certain hydrophilic interactions between residues from Bcl-X_L_ protein and BH3 peptides [30]. These interactions involve the Arg residue next to the highly conserved Leu residue in the BH3 peptide and the Glu residue present in the helix preceding the central hydrophobic helix of Bcl-X_L_ protein. Such interactions are suggested to be responsible for the high binding affinity of BH3 peptides of certain pro-apoptotic proteins and the results are supported by affinity studies.
- Spectroscopic and mutational studies have demonstrated a possible correlation between the BH3 peptides and their helical stability [31]. MD simulations of several Bcl-X_L_- and A1-binding BH3 peptides showed that the BH3 peptides that have high-binding affinities for either of the protein exhibited stable helical segments in aqueous medium [32], [33]. Analysis of the simulated structures revealed the reasons for the stable nature of the amphipathic BH3 peptide helices. Although clustering of hydrophobic residues destabilized the helices to some extent, capping interactions and stable hydrophilic interactions from the solvent-exposed hydrophilic side of the amphipathic BH3 helices helped to retain the helical nature of high-affinity BH3 peptides in the solvent. Such studies can aid in designing BH3 mutants and BH3 mimetics that can bind to the anti-apoptotic proteins with very high binding affinity.
- The last and the most important factor is the flexibility in the hydrophobic-binding region. Since Bcl-X_L_ is recognized by the BH3 region of several pro-apoptotic proteins [5], [19], [22], [31], [34], it is important that the hydrophobic groove must accommodate diverse BH3 sequences with some common features like conserved hydrophobic residues. Very few studies have looked into this aspect of the Bcl-2 proteins. Moreover, it is intriguing to note that such a large hydrophobic patch is exposed when the BH3 peptide is not bound to the protein. To our knowledge, the behavior of solvent exposed hydrophobic groove is not thoroughly explored. In this study, we have carried out molecular dynamics simulations of Bcl-X_L_ protein in the apo-form. We have also considered structures of Bcl-X_L_ complexes in which the bound BH3 peptides were removed (holo Bcl-X_L_). Our results from multiple MD simulations of Bcl-X_L_ in apo- and holo-forms show that the hydrophobic residues from Bcl-X_L_ which were interacting with the bound BH3 peptides in the structures of complexes interact among themselves in apo and holo structures. Accessible surface area calculations indicate that the exposed bulky hydrophobic residues in the hydrophobic groove are partially shielded by the loop connecting the BH3-containing helix and the next helix. Our simulations using twin-range cut-off exhibit destabilization of BH3-containing helix whereas simulations which used Particle Mesh Ewald (PME) have shown the same helix as stable. Experiments have indicated that the BH3-containing helix has to undergo functionally important conformational changes for homo- or hetero-dimerization [35], [36]. Unwinding of BH3-containing helix can also help in binding diverse BH3 ligands derived from pro-apoptotic Bcl-2 proteins. It is possible that the event of BH3-containing helix destabilization in PME simulations requires a longer time-scale (from several hundred nanoseconds to microseconds time scale).

## Materials and Methods

### Initial structures

The starting structure for apo-Bcl-X_L_ corresponds to the PDB ID 1PQ0 (Resolution: 2.2 Å) [37]. The initial structure of holo-Bcl-X_L_ was obtained from the structure of Bcl-X_L_/Bim complex (PDB ID: 1PQ1; resolution: 1.65 Å) by removing the bound Bim peptide. In holo structure, by removing the bound BH3 peptide, the previously buried hydrophobic groove is now exposed. MD simulations of holo structure will give an idea whether the behavior of exposed hydrophobic patch is similar to that observed in apo structure simulations. Moreover, one will be curious to know what happens to those hydrophobic residues which were originally interacting with the BH3 peptide ligand in the structure of the complex. Hence simulations were carried out on both apo- and holo-Bcl-X_L_ structures and the results are compared.

**Figure 1:**
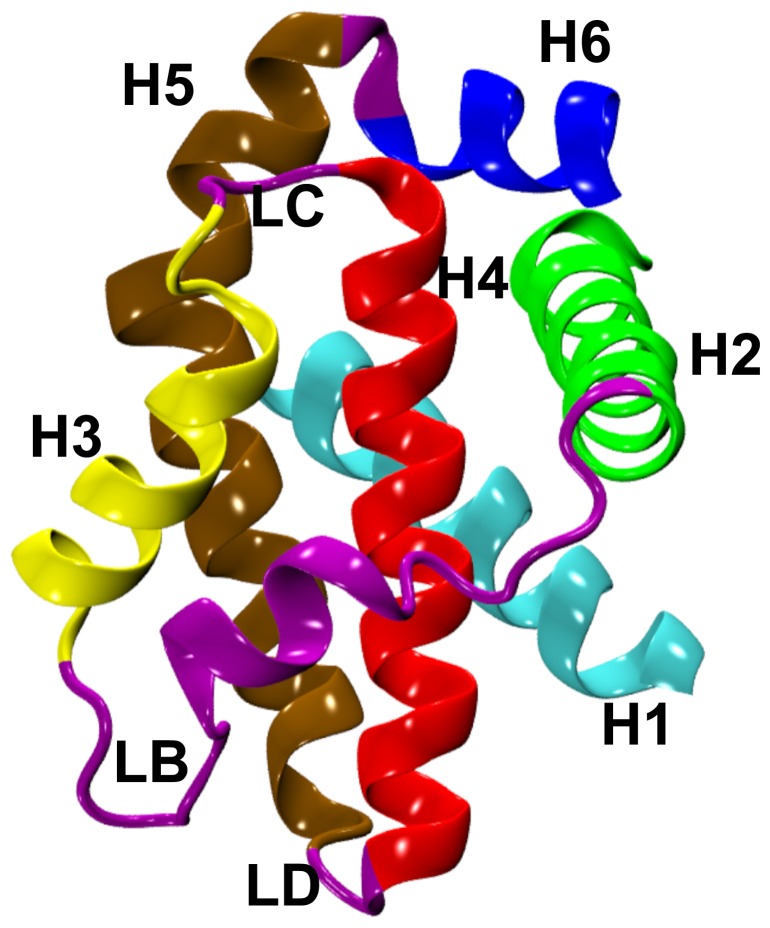
Ribbon representation of Bcl-X_L_ helical bundle structure. The major helices are labeled as H1 to H6 in this apo-structure (PDB ID: 1PQ0) and are shown in different colors. The loops (LB, LC and LD) connecting different helical segments are shown in purple color. The loop LA connecting helices H1 and H2 is not shown for clarity. Molecular plots in this figure and subsequent figures were generated using the VMD software [84].

It must be noted that in apo and holo structures, the disordered long loop linking the first two helices is not fully resolved and the structures were determined by deleting the C-terminal transmembrane domain [37]. The structure of the missing loop was built using the “Homology” module of InsightII suite of software (Accelrys Inc., San Diego, CA) by considering another structure of Bcl-X_L_ complex (PDB ID: 1G5J [31]) as template. Wherever side-chain atoms are not resolved in the experimental structures, they were constructed using the “Biopolymer” module of InsightII. Residue numbers of Bcl-X_L_ correspond to the sequence with UniProt [38] accession code Q64373. The ribbon diagram of a Bcl-X_L_ structure with its different helices is shown in Figure 1.

In the PDB structures, hydrogen atoms were built using the utility tool PDB2GMX available in the GROMACS modeling software version 3.2.1 [39], [40]. Only those hydrogens bonded to the polar atoms or aromatic carbon atoms were added. The N- and C-terminal residues were capped using the –NH_2_ and –COOH groups respectively. All titratable residues were assumed to have default protonation states. The starting structures were placed at the center of a cubic box whose size was determined such that the distance between the protein and the edge of the box was at least 10 Å. SPC [41] water molecules were used to solvate the Bcl-X_L_ protein using GENBOX tool in GROMACS. Energy minimization of solvated protein was carried out using steepest descent and conjugate gradient methods. Harmonic positional restraints were applied on protein heavy atoms during minimization.

### Simulation protocol

Eight independent simulations of the solvated and energy-minimized Bcl-X_L_ structures were carried out for a period of 25 to 55 ns using GROMOS96 43 a1 force field [42], [43]. The simulations differed in the starting structures (apo- or holo-Bcl-X_L_; see above), equilibration protocol (two different schemes) and in the treatment of long-range interactions (twin-range cut-off or PME methods). In the simulations, the bonds and angles of solvent molecules were constrained using SETTLE algorithm [44]. All the bonds involving the hydrogen atoms of the solute were constrained using LINCS algorithm [45]. Periodic boundary conditions were applied in all three directions. For all the simulations, the reference temperature and pressure were considered to be 300 K and 1 bar respectively. Simulation temperature and pressure were maintained using Berendsen's algorithm [46]. For temperature, a coupling constant of 0.01 ps during equilibration and 0.05 ps for the production run was used. The boundary pressure was maintained with a coupling constant of 1.0 ps. The solute and solvent temperatures were coupled separately. A time-step of 0.002 ps was used and the non-bonded list was updated every 10 steps.

### Two different treatments of long-range interactions

In simulations that used twin-range spherical cut-off, non-bonded interactions were evaluated using a cut-off of 10 and 18 Å. In PME simulations, the real space electrostatic and van der Waals interactions were evaluated with a spherical cut-off of 10 Å. A grid space of 1.5 Å was used with sixth order interpolation. The system was neutralized by adding 13 Na+ ions at random positions by replacing water molecules. The GENION tool in GROMACS was used for this purpose.

### Equilibration Scheme I

The system was first equilibrated for 1 ns with harmonic positional restraints applied on the heavy atoms of Bcl-X_L_ protein using NVT ensemble (constant number of atoms, volume and temperature). In the next stage, all restraints were removed and the system was equilibrated for another 1 ns using NPT (constant number of atoms, pressure and temperature) ensemble. After 2 ns of equilibration, the apo- and holo-Bcl-X_L_ structures were simulated for a period of 25 to 55 ns using the same NPT ensemble.

### Equilibration Scheme II

In the simulation of Bcl-X_L_ complexes, we have previously shown unwinding of helix H2 [30]. A similar observation was made in simulations in which the systems were equilibrated using Scheme I (see Results section). One can argue that such unwinding could be due to artifacts resulting from inadequate equilibration of the systems. Hence in this scheme, the equilibration protocol was modified. After the first 1 ns of equilibration in which harmonic positional restraints were applied on all heavy atoms of the protein, the next 1 ns equilibration was carried out in four stages with restraints imposed on helix H2 and its environment. In the first stage, 250 ps of equilibration run was carried out by imposing harmonic positional restraints on residue pairs from helix H2 and other helices (H1, H4 and H6) if these residues have at least one pair of heavy atoms with distance within 4 Å. In the next three stages each consisting of 250 ps equilibration run, positional restraints were imposed first on all heavy atoms of H2, then only on the backbone atoms of H2 and finally only on the Cα atoms of H2. The last 1 ns of equilibration was carried out without any restraints. Out of a total of 3 ns equilibration, the first 2 ns was performed using NVT ensemble. The last 1 ns of equilibration and the production run used NPT ensemble. The apo- and holo- structures were simulated for a period of 55 ns production run after the 3 ns equilibration. All the apo- and holo-Bcl-X_L_ simulations using two different equilibration schemes and two different treatments of long-range interactions are summarized in Table 1.

**Table 1:** Details of simulations of Bcl-X_L_ in apo/holo form.

Simulation	Equilibration scheme^c^	Total no. of atoms	Box size (Å^3^)	Simulation length
Apo-I^a^	Scheme-I	46,527	78.0 * 78.0 * 78.0	55 ns
Apo-II^a^	Scheme-II	46,527	78.0 * 78.0 * 78.0	55 ns
Holo-I^a^	Scheme-I	50,988	80.5 * 80.5 * 80.5	55 ns
Holo-II^a^	Scheme-II	50,988	80.5 * 80.5 * 80.5	55 ns
Apo-pme^b^	Scheme-I	46,501	78.0 * 78.0 * 78.0	55 ns
Holo-pme-I^b^	Scheme-I	50,962	80.5 * 80.5 * 80.5	50 ns
Holo-pme-II^b^	Scheme-I	73,153	90.5 * 90.5 * 90.5	25 ns
Holo-pme-III^b^	Scheme-I	100,081	101.2 * 101.2 * 101.2	25 ns

a Twin-range spherical cut-off was used to calculate the long-range interactions. See Methods for details.

b PME scheme was used to evaluate the long-range interactions. See Methods for details.

c See Methods section for details of two different equilibration schemes.

## Results

We have presented and compared the results of all eight simulations for both apo- and holo-Bcl-X_L_ systems. Average helical content of Bcl-X_L_, stability of individual helical segments, root mean square deviation (RMSD) with respect to the starting structure, solvent accessibility and interactions of hydrophobic residues in the hydrophobic groove are some of the analyses that have been carried out for different simulations.

### Stability of Bcl-X_L_ protein

#### Helical content of Bcl-X_L_

**Figure 2:**
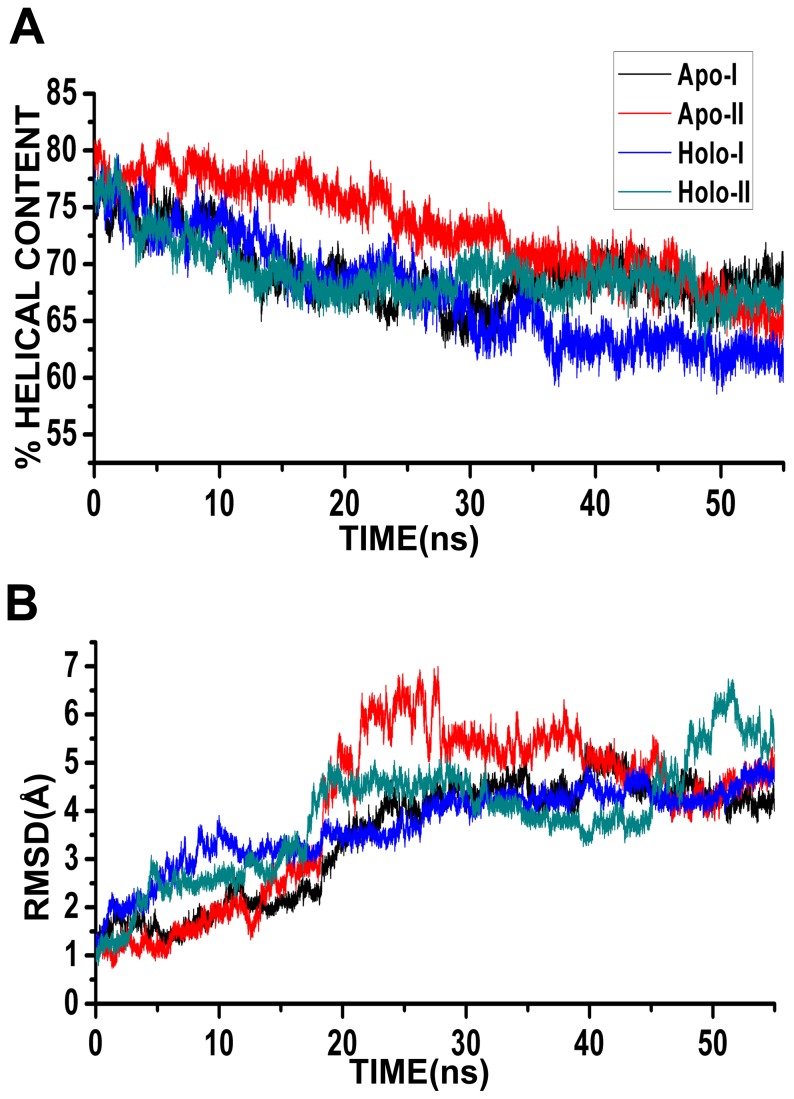
Stability of Bcl-X_L_ structure in twin-range cut-off simulations. MD trajectories of (A) percentage helical content and (B) RMSD profiles shown for the four simulations that used twin-range cut-off to evaluate the long-range interactions. Percentage helical content of each MD simulated structure was calculated as described in the Methods section. RMSD was calculated by considering the Cα atoms of all stable helical segments H1 to H6 (Table 2).

The average helical content of Bcl-X_L_ was calculated for each MD simulated structure as described in our earlier studies [30]. First, φ, ψ values were calculated for each residue and then a residue is considered to adopt right-handed α-helical conformation if the backbone dihedral angles satisfy the following criterion [47]

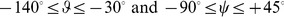
(1)Average helical content was calculated as the fraction of residues in Bcl-X_L_ having helical conformation. MD trajectories of average helical content for the four twin-range cut-off simulations are plotted in Figure 2A as the function of time. It is clear that the helical content of Bcl-X_L_ decreases to about 65 to 70% compared to almost 80% observed in the experimentally determined structures. The helical content is stabilized during the last 20 ns of production run. Compared to apo and holo simulations, Bcl-X_L_ retained a larger fraction of helical structure (75%) when it is bound to BH3 peptide of pro-apoptotic proteins as evident from the simulations of Bcl-X_L_ complexes [30].

**Figure 3:**
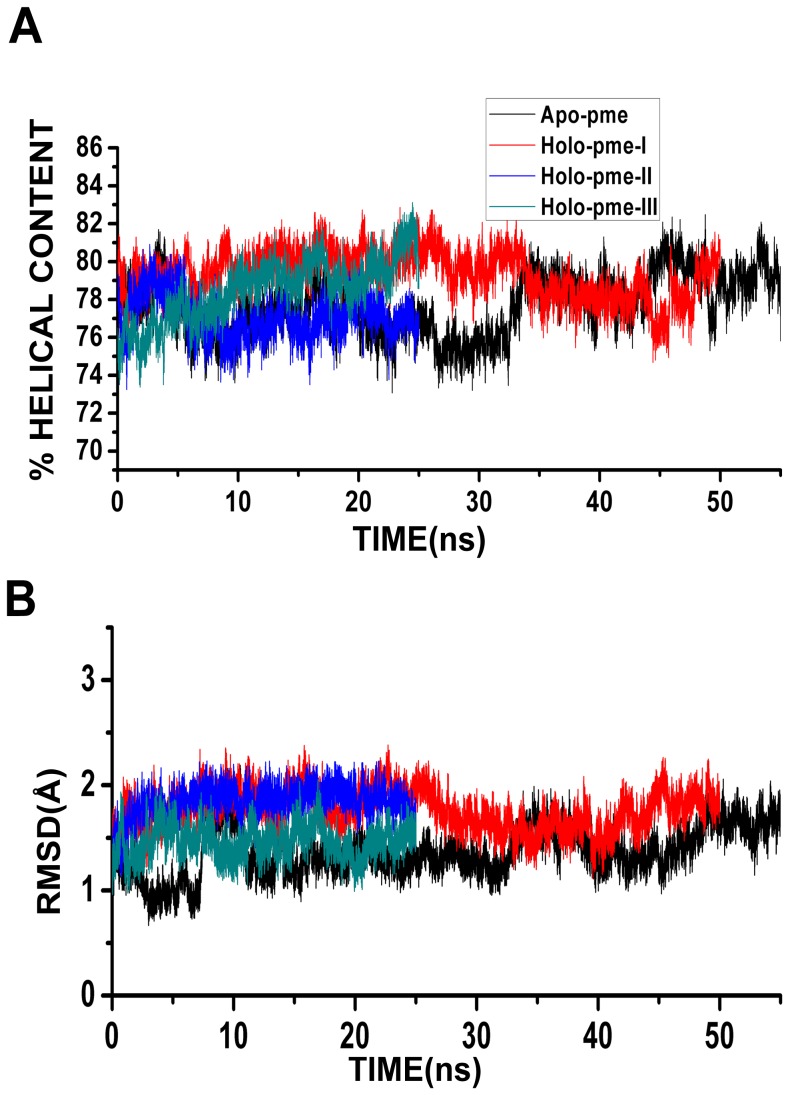
Stability of Bcl-X_L_ structure in PME simulations. MD trajectories of (A) percentage helical content and (B) RMSD profiles shown for the four simulations that used PME to treat the long-range interactions. Percentage helical content of each MD simulated structure was calculated as described in the Methods section. RMSD was calculated by considering the Cα atoms of all stable helical segments H1 to H6 (Table 2).

Helix content of each MD simulated structure was calculated and the MD trajectories of all four PME simulations are displayed in Figure 3A. It is clear that the helix content is maintained close to the experimentally determined structure (∼80%) in all the PME apo- and holo-Bcl-X_L_ simulations. This is in contrast to what was observed in twin-range cut-off simulations in which the protein lost about 10 to 15% of the total helical content in both apo and holo simulations. This demonstrates that the Bcl-X_L_ helix bundle structure is more stable in PME simulations compared to the twin-range cut-off simulations for the time period of simulation.

### Stability of individual helical segments

The decrease in the helical content of Bcl-X_L_ in twin-range cut-off simulations indicates that some of the helices might have lost the helical conformation either partially or fully. To identify and analyze the individual helical segments in the protein, we used the following definition. A segment in the polypeptide chain Bcl-X_L_ was considered helical if φ and ψ values of at least four consecutive residues must be in helical conformation. In other words, the φ and ψ values of at least four consecutive residues must satisfy equation (1). This definition was applied to identify the helical segments in the experimentally determined and MD simulated structures. We also defined a helical segment as stable if at least four consecutive residues maintain helical conformation for at least 80% of the simulation time.

Our criteria identified six helical segments designated as H1 to H6 in the experimentally determined structures (Table 2). The loop LB connecting H2 and H3 also contains a small helical segment. We also identified stable helical regions in the simulations Apo-I, Apo-II, Holo-I and Holo-II. We identified helical segments which are stable in at least two out of four twin-range cut-off simulations (Table 2). We noticed the formation of a small stable helical segment of just one turn in the loop LA connecting the helices H1 and H2. It is evident that there is a loss in helicity in helix H2 and this helix is broken in the middle (Figure S1). In fact in two simulations (Apo-I and Holo-II), this helix is almost completely destabilized. A similar observation was made in the simulations of three Bcl-X_L_ complexes although the unwinding was less dramatic in those cases [30]. This unwinding was also shown in a simulation using a different force field [48] with spherical cut-off [30].

**Table 2:** Stable helical regions in Bcl-X_L_ apo/holo twin-range cut-off simulations.

Helix	Experimental structures^a^^, ^ b	Stable helical regions^a^^, ^ d
H1	3–20	3–18
LA	-	24–27
H2	42–44, 85–100^c^	85–89, 96–100
LB	108–112	108–111
H3	120–133	120–131
H4	137–157	137–156
H5	159–185	160–183
H6	188–195	188–194

a The residue numbering is according to the Bcl-X_L_ sequence in UniProt database with the accession code Q64373.

b The helical regions common to the two Bcl-X_L_ crystal structures (PDB ID: 1PQ0 and 1PQ1) are reported. The definition of helical segment in the protein crystal structure is given in the Methods section.

c The loop region between H1 and H2 is not resolved in the experimentally determined crystal structures. The loop was built by homology modeling procedure with another Bcl-XL structure (PDB ID: 1G5J) as the template. In this model, the residue 44 is covalently linked to residue 85.

d These helical segments are stable in at least two out of four twin-range cut-off simulations.

We then examined the stability of helix H2 in all PME simulations and not surprisingly, this helix was found to be very stable (See Figure S1). This observation is unlike what was observed in the twin-range simulations in which significant portion of helix H2 lost the helical character.

As far as the other helices are concerned, with the exception of the C-terminal helix H6, other major helices are more or less intact throughout all the four twin-range cut-off simulations. The terminal regions of a polypeptide chain are usually flexible and in this case, the region after helix H6 is truncated. Hence, the instability in helix H6 was not surprising.

**Figure 4:**
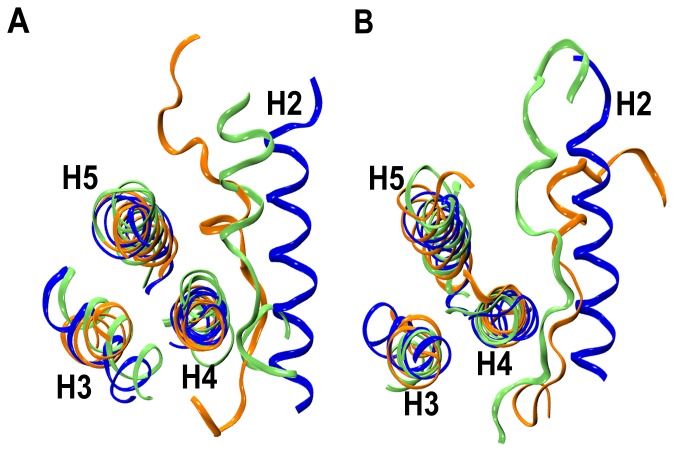
Unwinding of BH3-containing helix H2 in twin-range cut-off simulations. Superimposition of starting (blue) and MD simulated Bcl-X_L_ structures from (A) apo [Apo-I (orange) and Apo-II (green)] and (B) holo [Holo-I (orange) and Holo-II (green)] simulations. MD simulated structures were saved at the end of 55 ns production runs. The Cα-coordinates of the stable helical regions (Table 2) from helices H1, H3, H4 and H5 were considered for superposition. The helices H1 and H6 and the loops are not displayed for the sake of clarity.

**Figure 5:**
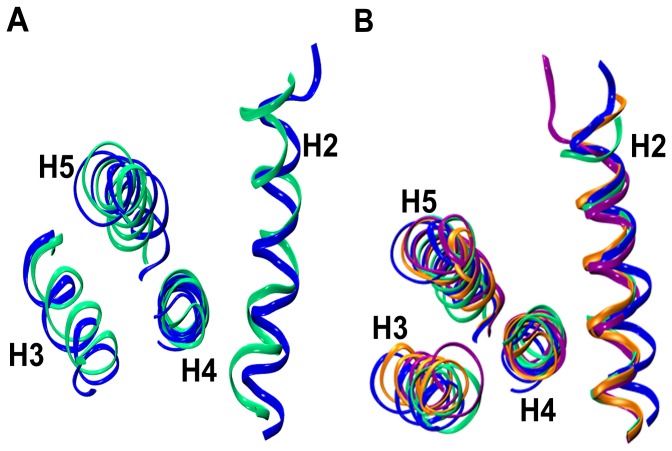
BH3-containing helix H2 is stable in PME simulations. Superimposition of starting (blue) and MD simulated Bcl-X_L_ structures from (A) apo [Apo-pme (green)] and (B) holo [Holo-pme-I (green), Holo-pme-II (orange) and Holo-pme-III (purple)] simulations. MD simulated structures were saved at the end production runs. The Cα-coordinates of the stable helical regions (Table 2) from helices H1, H3, H4 and H5 were considered for superposition. The helices H1 and H6 and the loops are not displayed for the sake of clarity.

There appears to be several reasons for the destabilization of helix H2 in twin-range cut-off simulations. In the complex simulations, when a similar observation was made, it was suggested that the destabilization of helix H2 could be due to the presence of a glycine residue (Gly 94), or presence of residue pairs which are either acidic [(E92, D95), (E92, E96), (D95, E98)] or basic (K87, R91). These residue pairs are separated by three to four residues [30]. In such an arrangement, the charged residues will be one above the other if they are present in an α-helix and as a result, these like-charged residue pairs will repel each other. A third factor was suggested to be the absence of stable inter-helix interactions involving helix H2 [30]. In Bcl-X_L_ apo/holo simulations as in complex simulations, we have analyzed all three factors (data not shown). Based on this analysis, we reached a similar conclusion in apo/holo Bcl-X_L_ simulations that all three factors individually or together could contribute to the destabilization of helix H2. MD simulated structures saved at the end of the production runs superimposed on the starting structure for the apo (Apo-I and Apo-II) and holo (Holo-I and Holo-II) simulations are shown in Figure 4A and 4B respectively. In PME simulations, the length of the simulations perhaps could be the reason that the unwinding has not been observed in helix H2. Longer simulations could have resulted in destabilization of the same helix. MD simulated structures superposed with the starting structure are shown in Figure 5 for the PME simulations.

### RMSD analysis

MD trajectories of RMSD (Root Mean Squared Deviation) calculated between the simulated structures and the starting structure are plotted in Figure 2B for the four twin-range cut-off simulations. Only C^α^ atoms of the residues from the six stable helical segments, H1 to H6 (Table 2), were considered for this purpose. At the end of 55 ns simulations, the structures deviated from the starting structures by close to 5 Å. This deviation is much larger, at least by 1.5 to 2 Å, compared to the Bcl-X_L_ complex simulations [30]. This indicates that the interacting BH3 peptides restrict the overall dynamics of the Bcl-X_L_ protein. In both apo and holo forms, Bcl-X_L_ does not have any constraints in the form of intermolecular interactions and the protein is relatively more flexible as evident from the RMSD analysis.

We next calculated RMSD of each MD simulated structure with respect to the starting structure for the PME simulations. As in the twin-range simulations, only the Cα atoms of the six major helical segments (H1 to H6; see Table 2) were considered for this purpose. Analysis of RMSD trajectories shows that all PME simulations exhibit a very low RMSD of 1.5 Å (Figure 3B). This again demonstrates that the protein has deviated less from the starting structures in all PME simulations.

### Interactions of solvent-exposed hydrophobic residues in the hydrophobic groove

While the hydrophobic groove is shielded from the solvent by the bound BH3 peptide in structures of complexes, it is exposed in apo-Bcl-X_L_. In general, exposure of a large hydrophobic patch in aqueous medium is not energetically favorable. Hence, it is intriguing to find out the behavior of this hydrophobic patch in the solvent-exposed state in both apo- and holo-Bcl-X_L_ structures. First, we specifically focused our attention on those hydrophobic residues which participated in interactions with the bound BH3 peptide in structures of complexes. We analyzed whether these residues now participate in interactions with other residues. For this purpose, we identified 174 interacting residue pairs from four experimentally determined structures; one apo-Bcl-X_L_ (PDB ID: 1PQ0) and three holo-Bcl-X_L_ structures (PDB IDs: 1BXL, 1G5J and 1PQ1; the bound BH3 peptides were removed from the structures of complexes). We define two residues as interacting residues if at least one pair of heavy atoms between the residues is within 4 Å. The interacting residue pairs occur between the six helices H1 to H6 as inter-helical interactions or between one of these helices and loops LB, LC or LD (Figure 1). We included these loops since they also contribute to the hydrophobic groove. The minimum distances between these residue pairs were followed throughout the simulations and analyzed for the last 20 ns of the production runs. An interaction is defined as stable if the minimum distance between at least one pair of heavy atoms is less than 4 Å for more than 50% of the analysis period in at least 2 out of 4 twin-range cut-off simulations. We have identified a total of 38 residue pairs which can be considered as participating in stable interactions. Majority of 38 interactions are observed in two out of three Bcl-X_L_ complex simulations [30] and are common with apo/holo simulations (data not shown). These interactions are mostly hydrophobic and occur in the interior of the helix bundle providing overall structural stability for the protein.

**Figure 6:**
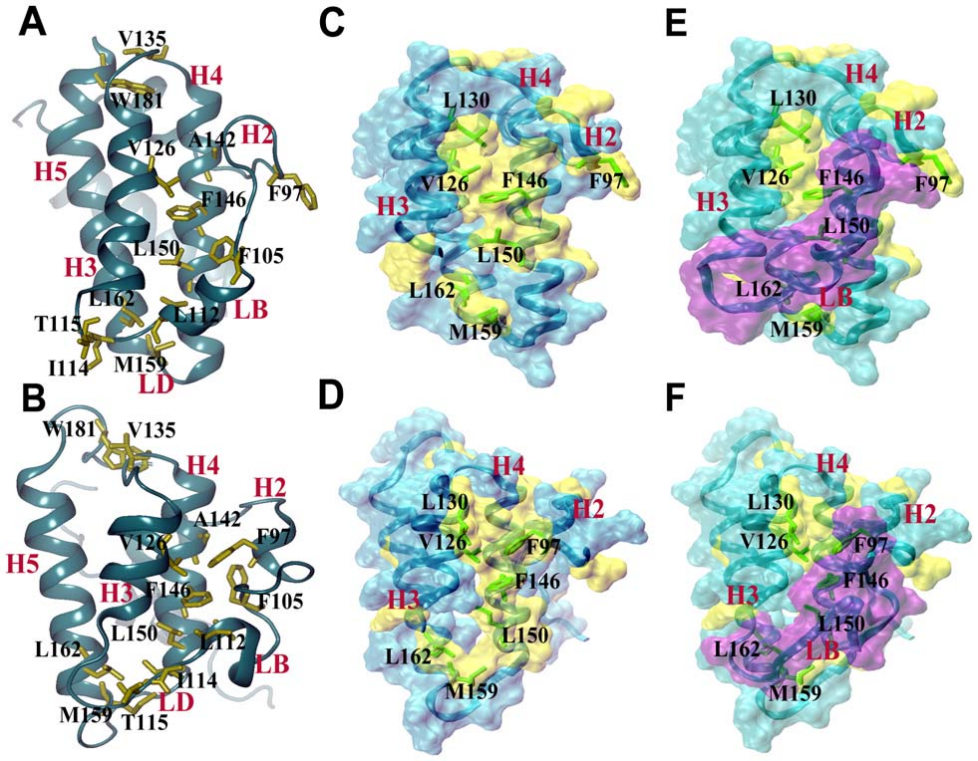
Interactions of hydrophobic residues in the hydrophobic groove and their accessible surface areas. Interactions among the hydrophobic residues in the hydrophobic groove are shown for (A) Apo-I and (B) Holo-I simulations. Helices and side-chains of hydrophobic residues are displayed in ribbon and stick representation respectively. Surface and ribbon representations of helices H2, H3, H4, H5 and loop LD (cyan) along with the hydrophobic residues from these regions (yellow) are shown for (C and E) Apo-I and (D and F) Holo-I simulations without loop LB (C and D) and with loop LB (E and F). Loop LB surface is represented in purple color in (E) and (F). The Bcl-X_L_ structures shown in this figures were saved at the end of 55 ns production runs from Apo-I and Holo-I simulations.

Eight stable interactions were identified only in the apo- and holo-Bcl-X_L_ simulations (Table 3). Five of them involve residue pairs in which at least one residue participated in stable interactions with a BH3 peptide in structures of complexes [30]. The residues from these residue pairs are from helices and loops that form the hydrophobic groove. This shows that in the absence of a bound BH3 peptide ligand, the exposed hydrophobic residue from the hydrophobic groove can compensate the energy penalty to some extent by interacting with another hydrophobic residue in the same hydrophobic groove. The remaining three stable interactions involve a residue in loop LB which is known to interact with the BH3 peptide [30]. All the eight stable interactions are shown in Figure 6A and 6B from Apo-I and Holo-I simulations. The same data for the other six simulations are provided in Figures S2, S3, S4. It is clear that these stable hydrophobic interactions which are absent in the Bcl-X_L_ complex simulations are spread throughout the hydrophobic groove from one end to the other end.

**Table 3:** Average minimum distance (in Å) for residue pairs involved in interactions^a^.

Residue pairs^b^	Interacting regions	Apo-I^c^	Apo-II^c^	Holo-I^c^	Holo-II^c^	Apo-pme	Holo-pme-I	Holo-pme-II	Holo-pme-III
**F97**…**A142**	H2, H4	5.62 (51)	3.91 (79)	3.86 (64)	3.52 (92)	3.55 (95)	3.22 (97)	3.29 (95)	3.21 (97)
**V126**…**F146**	H3, H4	3.59 (94)	3.96 (60)	3.70 (85)	3.87 (74)	3.99 (60)	3.50 (88)	3.30 (96)	3.34 (98)
**F105**…**F146**	LB, H4	3.89 (70)	3.95 (68)	3.55 (98)	6.73 (0)	3.73 (80)	2.64 (100)	2.60 (100)	2.61 (100)
**L112**…L150	LB, H4	3.65 (87)	4.03 (53)	3.45 (98)	8.60 (0)	3.86 (68)	6.65 (1)	3.84 (75)	3.61 (88)
V135…**W181**	LC, H5	3.28 (99)	4.01 (66)	3.16 (99)	3.23 (98)	3.65 (91)	2.92 (99)	3.23 (97)	2.72 (100)
I114…M159	LB, LD	4.33 (58)	5.20 (34)	4.05 (56)	3.48 (90)	4.30 (52)	3.48 (88)	6.08 (15)	3.59 (87)
I114…L162	LB, H5	3.94 (64)	4.13 (56)	6.02 (15)	3.81 (74)	3.80 (77)	3.76 (77)	4.47 (26)	4.21 (49)
T115…L162	LB, H5	6.15 (0)	3.65 (83)	4.01 (63)	4.17 (50)	3.34 (97)	3.86 (68)	6.46 (0)	4.72 (12)

a These interactions are stable in apo/holo Bcl-X_L_ simulations but not in the Bcl-X_L_ complex simulations [30].

b Residue shown in bold participate in stable interactions with the BH3 peptide ligands in Bcl-X_L_ complex simulations [30].

c The numbers in bracket represent the percentage time in which the minimum distance between the residue pairs was less than 4.0 Å during the analysis period.

### Solvent accessibility of hydrophobic residues in the hydrophobic groove

We calculated the solvent accessible surface area (SASA) of sixteen hydrophobic residues (A89, A93, F97, V126, L130, V135, V141, A142, F146, L150, M159, L162, W181, F191, L194 and Y195) that participate in the formation of hydrophobic groove and majority of these residues were in stable contact with at least one BH3 peptides (Bak, Bad and Bim) identified in our earlier simulations studies [30] of Bcl-X_L_ complexes. In the apo- and holo-Bcl-X_L_ structures, these residues are free, exposed and there is no bound ligand to interact. Since loop LB wraps around the binding BH3 peptides or ligands in the structures of complexes, SASA values for these hydrophobic residues were calculated with and without the loop LB (residues 101 to 119). This will give an idea of how much the loop LB contributes in burying the exposed hydrophobic residues. We also performed the same analysis for all the six hydrophobic residues (Y101, A104, F105, L108, L112 and I114) from the loop LB. The GROMACS (version 3.3) utility tool g_sas was used for this purpose. Average SASA values were calculated for the last 5 ns of the production run (Table 4) for the residues forming the hydrophobic groove. For loop LB hydrophobic residues, average SASA values have been reported for the first and the last 5 ns of the production run (Table 5).

**Table 4:** Average solvent accessible surface area (SASA in Å^2^)^a^ of hydrophobic residues in the hydrophobic groove which are significantly buried by the loop residues from the loop LB.

Residue (region)^b^	Apo-I^c^	Apo-II^c^	Holo-I^c^	Holo-II^c^	Apo-pme	Holo-pme-I	Holo-pme-II	Holo-pme-III
**F97** (H2)	79.3	62.7	12.9	38.8	10.0	38.3	44.1	28.7
	149.0	95.7	71.3	73.7	66.6	80.7	83.1	65.6
**V126** (H3)	42.0	4.5	1.6	8.7	19.6	40.0	42.2	28.9
	61.0	49.3	8.0	50.2	78.8	63.3	64.4	38.0
L130 (H3)	20.7	27.3	73.8	9.0	28.0	57.4	49.2	35.1
	23.6	43.5	96.6	22.2	75.9	62.1	50.7	42.2
**F146** (H4)	7.8	7.1	2.1	40.2	3.5	6.1	17.4	20.8
	58.1	82.4	70.0	77.9	77.3	53.9	66.5	76.2
**L150** (H4)	5.9	13.6	13.5	11.5	13.5	9.5	3.6	6.6
	62.9	65.2	87.3	61.4	59.3	51.1	51.8	61.5
**M159** (LD)	43.6	10.6	22.4	56.8	49.1	65.0	75.8	50.6
	87.3	20.5	50.1	98.4	88.0	103.0	110.8	91.4
**L162** (LD)	11.8	18.0	4.9	13.3	10.2	7.3	18.3	14.8
	69.5	85.9	65.2	61.3	68.5	63.3	44.7	68.2
Total	211.1	143.8	131.2	178.3	133.9	223.6	250.6	185.5
	511.4	442.5	448.5	445.5	514.4	477.4	472.0	443.1
Area buried by loop LB	300.3	298.7	317.3	267.2	380.5	253.8	221.4	257.6

a SASA was calculated for a given residue using the g_sas tool available in GROMACS version 3.3 [39], [40].

b Residues shown in bold participate in stable interactions with at least one BH3 peptide ligands in Bcl-X_L_ complex simulations [30]. For definition of secondary structure regions, see Table 1.

c For each residue, the top and bottom figures are the average SASA values calculated with and without loop LB respectively.

**Table 5:** Average solvent accessible surface area (SASA in Å^2^)^a^ of loop LB hydrophobic residues.

Residue in LB^b^	Apo-I^c^	Apo-II^c^	Holo-I^c^	Holo-II^c^	Apo-pme	Holo-pme-I	Holo-pme-II	Holo-pme-III
Y101	*133.8*	*89.2*	*111.6*	117.0	97.3	**91.3**	110.0	104.4
	*31.2*	*27.4*	*78.1*	48.1	95.8	**110.7**	112.3	104.3
A104	**39.1**	26.9	*44.1*	**57.4**	*32.1*	*39.8*	49.3	47.8
	**56.6**	20.0	*0.1*	**67.6**	*21.1*	*29.9*	47.0	55.4
**F105**	*57.0*	**29.4**	**34.8**	**30.5**	**25.0**	21.7	28.2	25.0
	*31.6*	**107.4**	**43.9**	**61.6**	**39.0**	21.6	28.8	24.8
L108	*30.9*	17.2	**7.8**	**5.1**	*24.2*	14.8	18.8	10.1
	*20.3*	26.7	**84.3**	**15.2**	*6.9*	14.5	18.3	15.8
**L112**	12.5	*21.1*	*29.6*	50.1	**11.8**	65.6	10.9	*44.5*
	15.9	*7.1*	*4.7*	52.6	**23.5**	58.6	6.0	*24.5*
**I114**	**34.4**	**29.1**	**22.1**	2.0	43.8	17.7	9.9	**24.6**
	**87.5**	**51.6**	**45.4**	7.6	30.4	25.7	13.4	**100.2**

a SASA was calculated for a given residue using the g_sas tool available in GROMACS version 3.3 [39], [40].

b Residues shown in bold participate in stable interactions with at least one of the BH3 peptide ligands in Bcl-X_L_ complex simulations [30].

c For each residue, the top and bottom numbers represent respectively the average SASA values calculated for the first and the last 5 ns of production runs. If the residues are buried during the simulation, then the values are shown in italics and underlined. SASA values of exposed residues are shown in bold.

We found that average SASA values of 8 out of 16 residues (A89, A93, V135, V141, W181, F191, L194 and Y195) are not affected by loop LB (data not shown). These residues are present in the unwound region of helix H2 or close to other loop regions. Accessibility of the seven residues (F97, V126, L130, F146, L150, M159 and L162) is significantly affected by the presence of loop LB. These are bulky hydrophobic residues and the loop LB buries up to 75 Å^2^ surface area for each of these seven residues. They are present in the regions (helices H2, H3 and H4 and loop LD connecting helices H4 and H5) that significantly contribute to the formation of hydrophobic groove (Figure 6C and 6D). It is also interesting to note that six out of seven of these residues also participate in stable hydrophobic interactions only in apo- and holo-Bcl-X_L_ simulations (Table 3). In all four simulations, presence of loop LB helps to bury a total of 221 to 380 Å^2^ in these seven hydrophobic residues (Figure 6E and 6F; also see Figures S2, S3, S4).

We next analyzed the SASA values of all the hydrophobic residues from loop LB to see whether a specific pattern is observed in burying or exposing the hydrophobic residues across the eight simulations. In all the twin-range cut-off simulations, the Y101 residue which was initially exposed to the solvent was buried at the end of 55 ns production run (Table 5). Apart from this observation, no definite trend was found across all simulations. However, it is observed that change in the SASA value of hydrophobic residues which result in exposure or burial is more often observed in twin-range cut-off simulations. Burial or exposure of hydrophobic residues during the course of PME simulations do not occur frequently. This shows that the dynamics of loop LB in burying the hydrophobic groove is different between the PME simulations and those simulations which used twin-range cut-off.

In summary, the hydrophobic residues in the hydrophobic groove, which were participating in interactions with BH3 peptide ligands, take part in stable interactions among themselves. The dynamic nature of loop LB connecting the helices H2 and H3 helps to bury up to 250 to 380 Å 2 surface area of bulky hydrophobic residues from exposure to the solvent. Both observations help to explain how the exposed hydrophobic groove remains stable in solvent-exposed environment.

## Discussion

### Functional significance of helix H2 unwinding

One of the significant results of apo- and holo-Bcl-X_L_ simulations using twin-range cut-off is the dramatic unwinding of helix H2 which was also observed in the Bcl-X_L_ complex simulations [30]. It is important to note that helix H2 contains the functionally important BH3 domain and such unwinding is not observed in other major helices of Bcl-X_L_. This observation could have functional significance in two respects and the first one is related to the binding of diverse BH3 peptide ligands to the hydrophobic groove of Bcl-X_L_. The unfolding of helix H2 can provide flexibility to the hydrophobic groove and hence Bcl-X_L_ can bind to the BH3 regions of different pro-apoptotic proteins with different affinities [5], [22]. This is evident from the fact that Bcl-X_L_ interacts with the BH3 peptides of Bak, Bad and Bim pro-apoptotic proteins and the affinities differ from 0.6 nM to 340 nM [19], [21], [22]. Structures of Bcl-X_L_ in complex with mutant BH3 peptides and BH3 peptidomimetics revealed structural transitions that might take place in the BH3-binding groove [23], [49]. Structural plasticity of hydrophobic groove has been demonstrated in other Bcl-2 family structures in complex with BH3 domains of different pro-apoptotic proteins [50]. Plasticity in ligand-binding region has been recognized in other diverse proteins also [51], [52], [53], [54]. Thus the unwinding of H2 can be related to the protein's ability to bind different BH3 domains with differential affinities.

The second aspect of helix H2 unwinding may have implications in the formation of homo/hetero dimers of Bcl-2 members. The diverse Bcl-2 proteins are known to adopt the same helical fold [18], [55] and hence the hydrophobic face of the amphipathic BH3 region which is contained in helix H2 will be buried in this fold. In order for the BH3 region of these pro-apoptotic proteins to bind in the hydrophobic groove of their anti-apoptotic partners, the BH3 region has to be exposed. The exposure of BH3 region requires conformational change of helix H2. One suggested change in earlier studies was rotation of helix H2 to expose its hydrophobic surface [19]. However since helix H2 forms part of the protein's hydrophobic groove, a simple rotation with helical structure intact requires breaking of hydrophobic contacts with the rest of the protein and such conformational change may not be energetically feasible. As observed in earlier complex simulations [30] and the present apo- and holo-Bcl-X_L_ simulations, unwinding of helix H2 and later reforming the helix with hydrophobic surface exposed for binding with the hydrophobic groove may be one way that can lead to the protein-protein interactions. Formation of homo- and hetero-dimers has been reported for several Bcl-2 members including Bcl-X_L_ and the interaction occurs when BH3 domain of one protein binds to the hydrophobic groove of another protein [56], [57], [58]. Several studies have suggested that exposure of BH3 domain is a crucial step for homo- and hetero-dimerization of Bcl-2 proteins. In many studies that investigated dimerization of the pro-apoptotic proteins Bax and Bak, exposure of BH3 domain seems to be critical [35], [36], [56]. For example, studies on Bak mutants altering its BH3 domain or its hydrophobic groove clearly influenced its ability to form oligomers and this step is required for its pro-apoptotic function [35]. This study demonstrated that exposure of BH3 domain of Bak is an essential early step for its interaction with the hydrophobic groove. The present simulation studies suggest that such exposure of BH3 domains can occur first by unwinding the BH3-containing helix H2 and later reforming the helix by exposing its hydrophobic side to the hydrophobic binding groove. Hence, unwinding of helix H2 can either provide structural plasticity to the hydrophobic groove by allowing diverse BH3 peptides to bind and/or can be part of the process to expose the BH3 domains that can facilitate the formation of homodimers or heterodimers. Either way, this event assumes major functional significance in the apoptotic pathway.

As far as the PME simulations are concerned, unwinding of helix H2 is not observed perhaps due to the fact that the lengths of the simulations are short. A detailed discussion comparing the results of PME and twin-range cut-off simulations is given in a separate section (see below).

### Dual role of flexible loop LB

**Figure 7:**
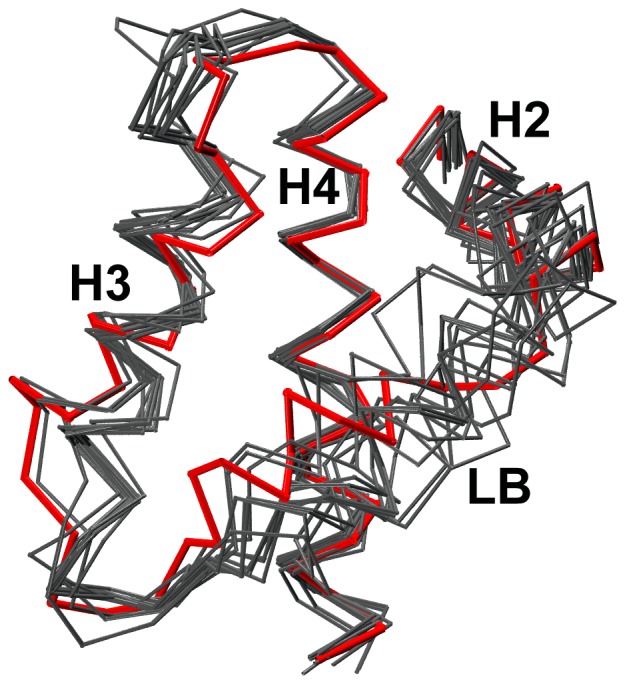
Flexible loop LB in structures of different Bcl-X_L_ complexes. Superimposition of 13 experimentally determined structures of Bcl-X_L_ complexes (black) on the apo-Bcl-X_L_ structure (PDB ID: 1PQ0 shown in red). Although more structures of complexes are available, we have chosen those complexes in which the ligands are unique. The Cα-atoms of helices H2, H3 and H4 of structures were superposed on the same helical segments of apo structure. This highlights the conformational heterogeneity of loop LB which connects H2 and H3. The PDB IDs of the structures of Bcl-X_L_ complexes are 1BXL, 1G5J, 1PQ1, 1YSG, 1YSI, 2P1L, 2YJ1, 2YXJ, 3FDM, 3INQ, 3PL7, 3QKD and 3R85.

The stability of exposed hydrophobic groove can be explained by two factors: (i) interactions among the hydrophobic residues in the groove and (ii) burial of hydrophobic amino acids by the residues from loop LB. Interactions between exposed hydrophobic residues is akin to hydrophobic collapse when a protein is on the way to its folding process trying to shield its hydrophobic residues from solvent exposure. Hydrophobic residues are also protected from the solvent by loop LB. This loop connects the BH3-containing helix H2 with the next helix H3 and it wraps around the hydrophobic groove (Figure 1). In the apo-Bcl-X_L_ structure, a short stretch of this loop assumes helical conformation while in the Bcl-X_L_ structures in complex with pro-apoptotic BH3 peptides, it essentially adopts a random conformation. It has been shown that the loop LB has stable interactions with the bound BH3 peptides [30]. The present simulation studies clearly demonstrate that this loop plays a major role in shielding the hydrophobic residues in the hydrophobic groove of Bcl-X_L_. The flexible and dynamic nature of this loop is evident by comparing the apo-Bcl-X_L_ structure with the structures of other Bcl-2 members, structures of Bcl-X_L_ complexes and also the mutant Bcl-X_L_ structures [19], [21], [31], [37], [59], [60], [61]. We have identified 13 structures of Bcl-X_L_ complexes in PDB in which the bound molecules are BH3 peptides or other organic ligands. Superposition of these structures (Figure 7) clearly demonstrates that loop LB displays conformational heterogeneity to accommodate and to have strong interactions with the ligands. In the absence of any bound ligands, this loop helps burying 250 to 380 Å^2^ surface area of bulky hydrophobic residues when all eight simulations are considered. The burial and exposure of loop LB residues is not uniform across all simulations. This indicates that loop LB is constantly sampling many conformations to optimally bury as many hydrophobic residues in the hydrophobic groove as possible. Thus loop LB plays a pivotal role in stabilizing the exposed hydrophobic residues in the hydrophobic groove in the absence of any bound ligand. The role of loop LB residues in conferring selectivity and higher affinities to different Bcl-X_L_ ligands has to be explored further.

### Twin-range vs PME simulations

Although the twin-range cut-off of 10 to 18 Å is long enough to include most of long-range interactions, one can always argue that the long-range interactions beyond 18 Å may still be not negligible. In such a case, the neglected long-range interactions could have influenced the properties and behavior of Bcl-X_L_ protein. Since the development of Ewald-based methods in early 1990s [62], [63], [64], they have become one of the most popular and widely used methods to calculate the long-range interactions in molecular simulations. Hence in this study, we have also simulated both apo- and holo-Bcl-X_L_ in which particle mesh Ewald (PME) scheme was used in calculating the long range interactions. Four simulations (Apo-pme-I, Holo-pme-I, Holo-pme-II and Holo-pme-III) have been carried out as described in the Methods sections and summarized in Table 1. These simulations differed in the initial structure (apo or holo) and the size of the box. In all four simulations, the same equilibration scheme (Scheme I) was used. The systems were simulated for a period of 25 to 55 ns.

**Figure 8:**
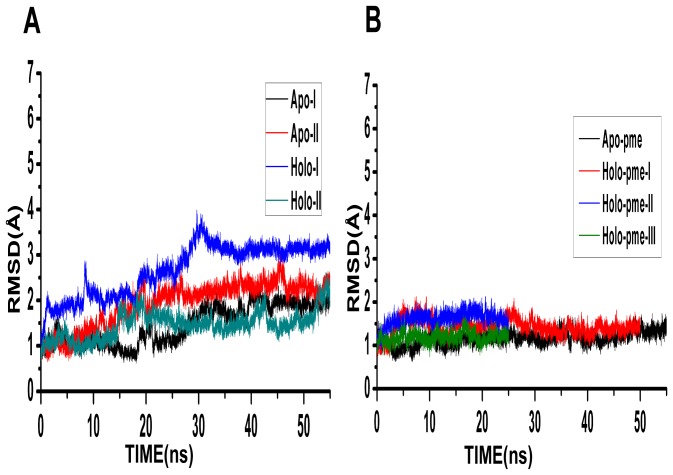
Comparison of two sets of simulations using RMSD analysis. Comparison of MD trajectories of RMSD values calculated for simulations that used (A) twin-range cut-off and (B) PME to calculate the long-range interactions. Only the helices H1, H3, H4 and H5 were considered for calculating RMSD between each MD simulated structure and the starting Bcl-X_L_ structure. Helix H2 and the C-terminal helix H6 were excluded in this analysis. Compare this figure with Figure 2B and Figure 3B.

When we compared the set of simulations that used twin-range-cut-off with the PME simulations, the most significant difference between the two sets is the helix H2 unwinding in the former while the same helix is very stable in the latter set of simulations. This gives rise to a set of questions. Is such an unwinding due to the neglect of long-range interactions in twin-range cut-off simulations? If it is so, why do most of the other major helices in apo- and holo-Bcl-X_L_ remain stable? Is this the only difference between the twin-range cut-off simulations and PME simulations? To answer the above questions, we first calculated the RMSD profiles of all the simulations with only helices H1, H3, H4 and H5 and the MD trajectories were compared between the two sets of simulations (Figure 8A and 8B). It is very clear that the RMSD profiles are very similar for the two sets of simulations if helix H2 and the C-terminal H6 are excluded from the analysis. This also accounts for the difference observed in the overall Bcl-X_L_ helical content between twin-range cut-off and PME simulations.

Molecular simulations using PME scheme to calculate long-range interactions have been compared with other methods like reaction field, twin-range cut-off and continuum models [65], [66], [67], [68], [69], [70], [71], [72], [73], [74], [75]. While these earlier studies reported less conformational sampling and reduced flexibility in PME simulations, several recent simulation studies have used PME and investigated flexible regions and conformational changes in diverse proteins [76], [77], [78], [79]. Hence, the highly stable helix H2 in apo- and holo-forms in the set of PME simulations in the present study indicates that the length of the simulations is not sufficient to observe the H2 destabilization. The time-period of present simulations, 25 to 55 ns, is perhaps not long enough for the helix H2 to unwind. This is further corroborated by a recent study of Bcl-X_L_ using Amber 99SB force field [80] and PME scheme. This study by Yang and Wang [81] reported only minor backbone changes for the apo-Bcl-X_L_ in water during the 32 ns simulation. Only in the presence of isopropanal cosolvent molecules, large conformational changes were observed in specific regions.

**Figure 9:**
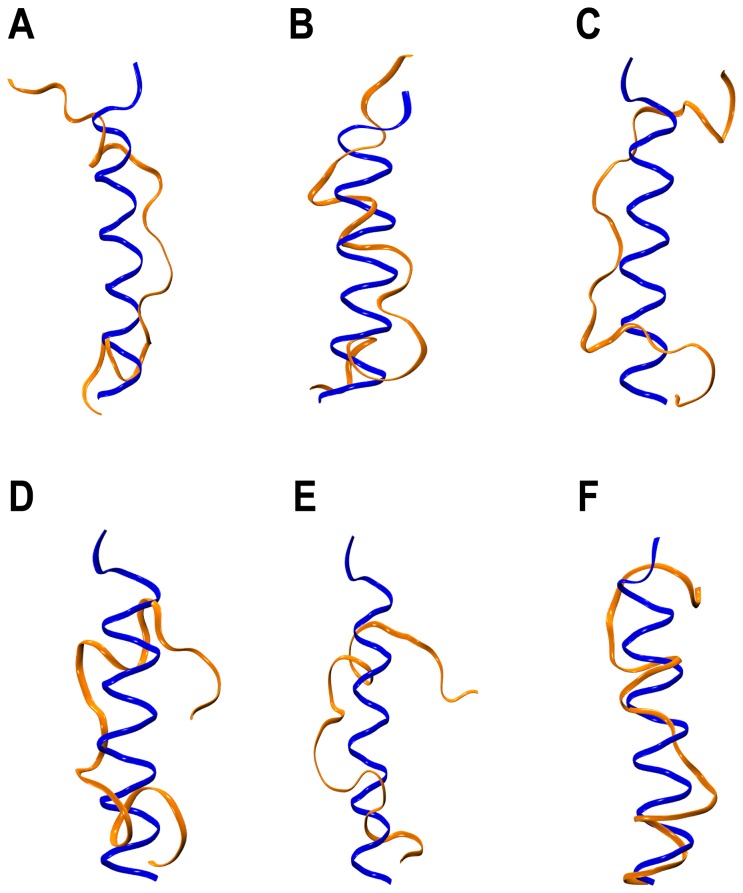
Helix H2 stability in the additional simulations. Superposition helix H2 from the starting structure (blue) and the structures saved at the end of the production runs (orange) from (A) Apo-cut-off-I, (B) Apo-cut-off-II, (C) Apo-cut-off-III, (D) Apo-H6-Extended-I, (E) Apo-H6-Extended-II and (F) Apo-H6-Extended-III simulations.

To further investigate the influence of the scheme that is used to calculate the long-range interactions on the helix H2 stability, we carried out six additional simulations (Table 6) using GROMACS version 4.5.5 [82]. Three simulations used longer twin-range cut-off. In the other three simulations, the truncated helix H6 was extended using the structure of Bcl-X_L_ complex (PDB ID: 1G5J) as a template. Within 10 to 35 ns, unwinding of helix H2 was observed in all the simulations (Figure 9). The choice of simulation conditions can affect the dynamics of the system as was demonstrated recently [83]. Hence, in the set of PME simulations, the dynamic behavior of Bcl-X_L_ showed differences with the twin-range cut-off simulations and we believe that a longer PME simulation would eventually produce a similar destabilization of helix H2.

**Table 6:** Details of additional Bcl-X_L_ simulations.

Simulation^a^	Remarks	Production run
Apo-cutoff-I	Twin-range cut-off: 12 to 20 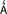	30 ns
Apo-cutoff-II	Twin-range cut-off: 14 to 22 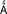	25 ns
Apo-cutoff-III	Twin-range cut-off: 16 to 24 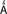	15 ns
Apo-H6-extended-I	Helix H6 was extended by modeling the residues 197 to 211^b^	15 ns
Apo-H6-extended-II	Simulation was started with different initial velocities using Apo-H6-extended-I	10 ns
Apo-H6-extended-III	Positional restraints were applied on heavy atoms of helix H6^c^	10 ns

a In all listed simulations, Scheme I equilibration protocol was followed. All other simulation parameters are provided in the Methods section.

b Bcl-X_L_ structure with PDB ID 1G5J was used as a template to model the extended helix H6. Modeller ver 9.11 [85] was used to model the longer helix H6.

c NVT ensemble was used.

## Conclusions

The anti-apoptotic Bcl-X_L_ protein is an attractive target for anti-cancer drugs. The pronounced hydrophobic groove formed by the helix bundle structure is the ligand-binding region and the Bcl-X_L_ inhibitors are developed keeping the physical and chemical nature of this groove in mind. In the absence of any bound ligand, the present simulation study investigated the behavior of the solvent-exposed hydrophobic groove. Eight independent simulations of Bcl-X_L_ in apo and holo form were carried out. These simulations used either twin-range cut-off or PME for calculating the long-range interactions. All the twin-range cut-off simulations exhibited destabilization of the BH3 domain-containing helix H2. However, the other major helices with the exception of C-terminal helix H6 were stable. At this point, it is speculated that the failure of PME simulations to destabilize the helix H2 may simply be due to the length of simulations. If the PME simulations are extended further, it is possible to detect the conformational changes associated with helix H2. Based on several experimental results, the unwinding of helix H2 can be linked to the plasticity of the hydrophobic groove which enables the Bcl-X_L_ protein to bind to different BH3 ligands with differential affinities. Helix H2 destabilization can also be connected to the formation of homo- or hetero-dimers of Bcl-2 proteins. Since helix H2 contains BH3 domain, it has to undergo conformational changes to expose the buried hydrophobic side of BH3 domain. Hence the loss of helical character for H2 seems to have functional significance. Results of simulation studies show that the exposed hydrophobic residues from the groove interact among themselves while in the structures of complexes, most of them were involved in interacting with the BH3 peptide ligands. The solvent accessible surface areas of these residues are significantly buried by the loop LB connecting the helices H2 and H3. This explains how the predominantly hydrophobic groove remains stable when exposed to the solvent. Understanding of the plasticity of hydrophobic groove and the dynamics of loop LB reported in this study can help in the design of inhibitor molecules that will be highly specific to Bcl-X_L_ protein.

## Supporting Information

Figure S1 **DSSP Plots of helix H2 of Bcl-X_L_**. DSSP plots of helix H2 for all 8 simulations using two different schemes to calculate long-range interactions. (A) Apo-I, (B) Apo-II, (C) Holo-I, (D) Holo-II, (E) Apo-pme, (F) Holo-pme-I, (G) Holo-pme-II and (H) Holo-pme-III.
(JPG)

Figure S2 **Hydrophobic residues in the hydrophobic cleft: Interactions and accessible surface areas in Apo-II and Holo-II simulations.** Interactions among the hydrophobic residues in the hydrophobic groove are shown for (A) Apo-II and (B) Holo-II simulations. Helices and side-chains of hydrophobic residues are displayed in ribbon and stick representation respectively. Surface and ribbon representations of helices H2, H3, H4, H5 and loop LD (cyan) along with the hydrophobic residues from these regions (yellow) are shown for (C and E) Apo-II and (D and F) Holo-II simulations without loop LB (C and D) and with loop LB (E and F). Loop LB surface is represented in purple color in (E) and (F). The Bcl-X_L_ structures shown in this figures were saved at the end of 55 ns production runs from Apo-II and Holo-II simulations.
(JPG)

Figure S3 **Hydrophobic residues in the hydrophobic cleft: Interactions and accessible surface areas in Apo-pme and Holo-pme-I simulations.** Interactions among the hydrophobic residues in the hydrophobic groove are shown for (A) Apo-pme and (B) Holo-pme-I simulations. Helices and side-chains of hydrophobic residues are displayed in ribbon and stick representation respectively. Surface and ribbon representations of helices H2, H3, H4, H5 and loop LD (cyan) along with the hydrophobic residues from these regions (yellow) are shown for (C and E) Apo-pme and (D and F) Holo-pme-I simulations without loop LB (C and D) and with loop LB (E and F). Loop LB surface is represented in purple color in (E) and (F). The Bcl-X_L_ structures shown in this figures were saved at the end of 55 ns production run from Apo-pme and 50 ns production run from Holo-pme-I simulation.
(JPG)

Figure S4 **Hydrophobic residues in the hydrophobic cleft: Interactions and accessible surface areas in Holo-pme-II and Holo-pme-III simulations.** Interactions among the hydrophobic residues in the hydrophobic groove are shown for (A) Holo-pme-II and (B) Holo-pme-III simulations. Helices and side-chains of hydrophobic residues are displayed in ribbon and stick representation respectively. Surface and ribbon representations of helices H2, H3, H4, H5 and loop LD (cyan) along with the hydrophobic residues from these regions (yellow) are shown for (C and E) Holo-pme-II and (D and F) Holo-pme-III simulations without loop LB (C and D) and with loop LB (E and F). Loop LB surface is represented in purple color in (E) and (F). The Bcl-X_L_ structures shown in this figures were saved at the end of 25 ns production runs from Holo-pme-II and Holo-pme-III simulations.
(JPG)
