# Supplementary figures and images for: Behavior of Solvent-Exposed Hydrophobic Groove in the Anti-Apoptotic Bcl-XL Protein: Clues for Its Ability to Bind Diverse BH3 Ligands from MD Simulations
Source: PLoS One. 2013 Feb 28;8(2):e54397. doi: 10.1371/journal.pone.0054397 (PMC3585337; doi:10.1371/journal.pone.0054397)

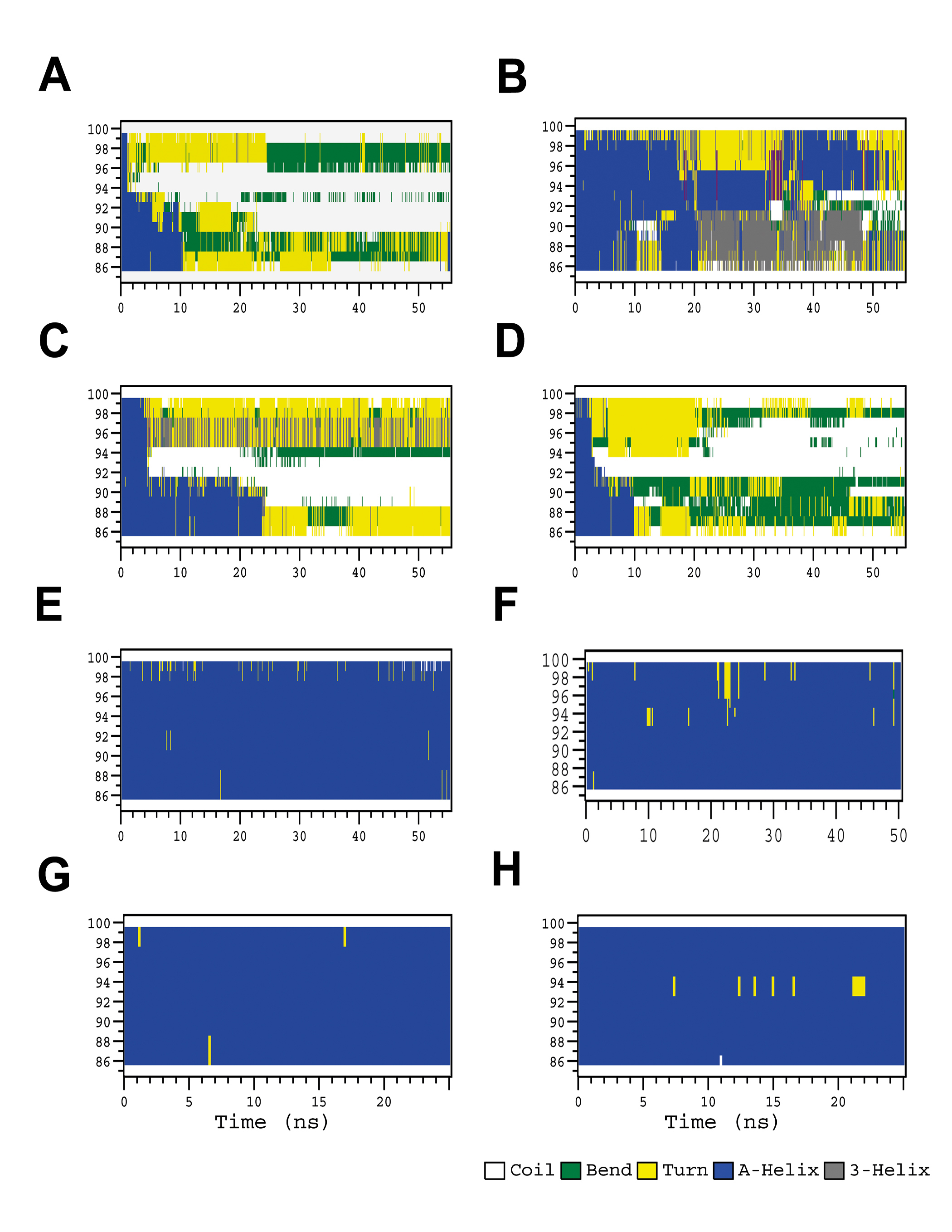

Supplement: Figure S1 — DSSP Plots of helix H2 of Bcl-XL. DSSP plots of helix H2 for all 8 simulations using two different schemes to calculate long-range interactions. (A) Apo-I, (B) Apo-II, (C) Holo-I, (D) Holo-II, (E) Apo-pme, (F) Holo-pme-I, (G) Holo-pme-II and (H) Holo-pme-III. (JPG) [file pone.0054397.s001.jpg]

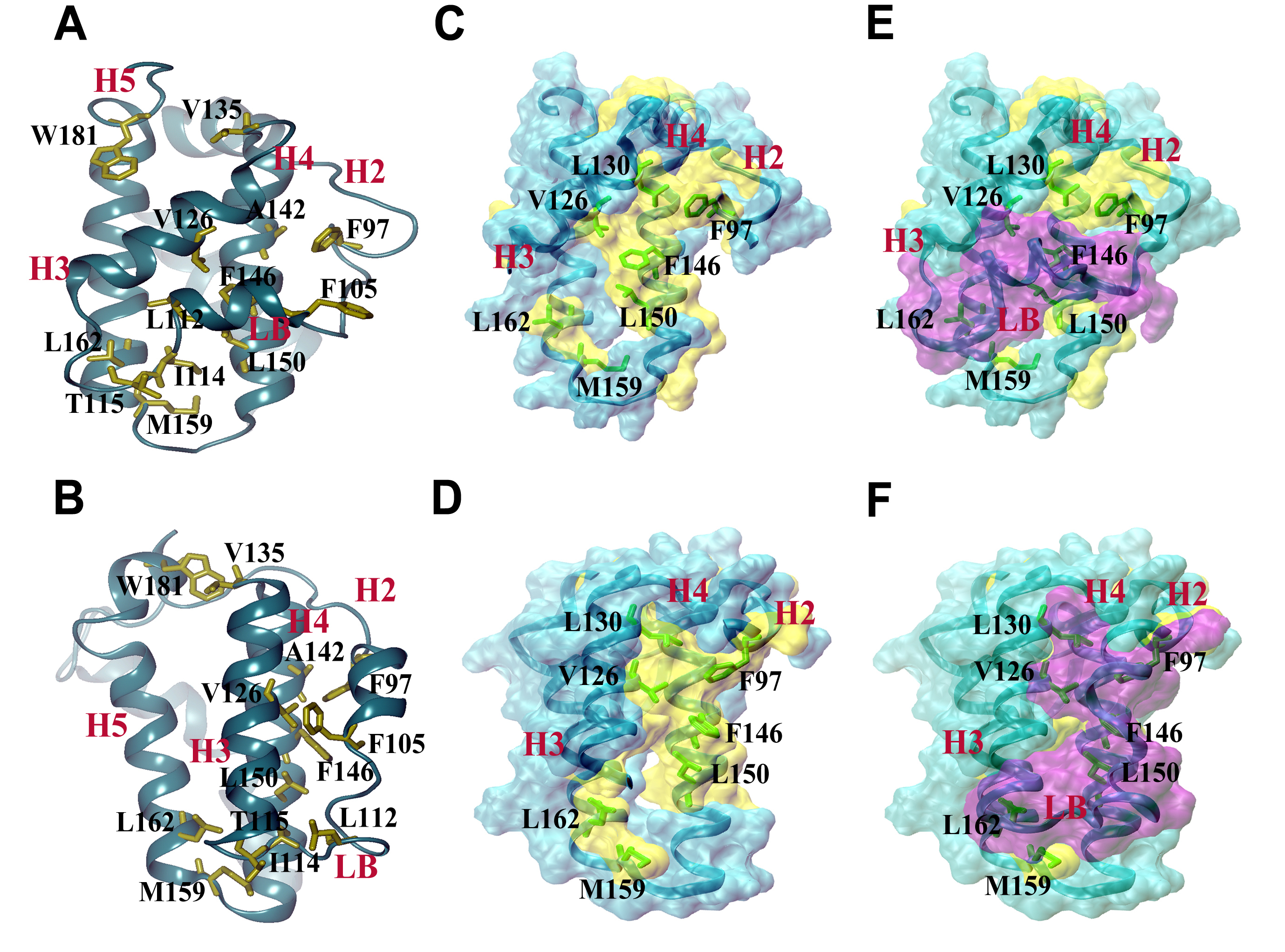

Supplement: Figure S2 — Hydrophobic residues in the hydrophobic cleft: Interactions and accessible surface areas in Apo-II and Holo-II simulations. Interactions among the hydrophobic residues in the hydrophobic groove are shown for (A) Apo-II and (B) Holo-II simulations. Helices and side-chains of hydrophobic residues are displayed in ribbon and stick representation respectively. Surface and ribbon representations of helices H2, H3, H4, H5 and loop LD (cyan) along with the hydrophobic residues from these regions (yellow) are shown for (C and E) Apo-II and (D and F) Holo-II simulations without loop LB (C and D) and with loop LB (E and F). Loop LB surface is represented in purple color in (E) and (F). The Bcl-XL structures shown in this figures were saved at the end of 55 ns production runs from Apo-II and Holo-II simulations. (JPG) [file pone.0054397.s002.jpg]

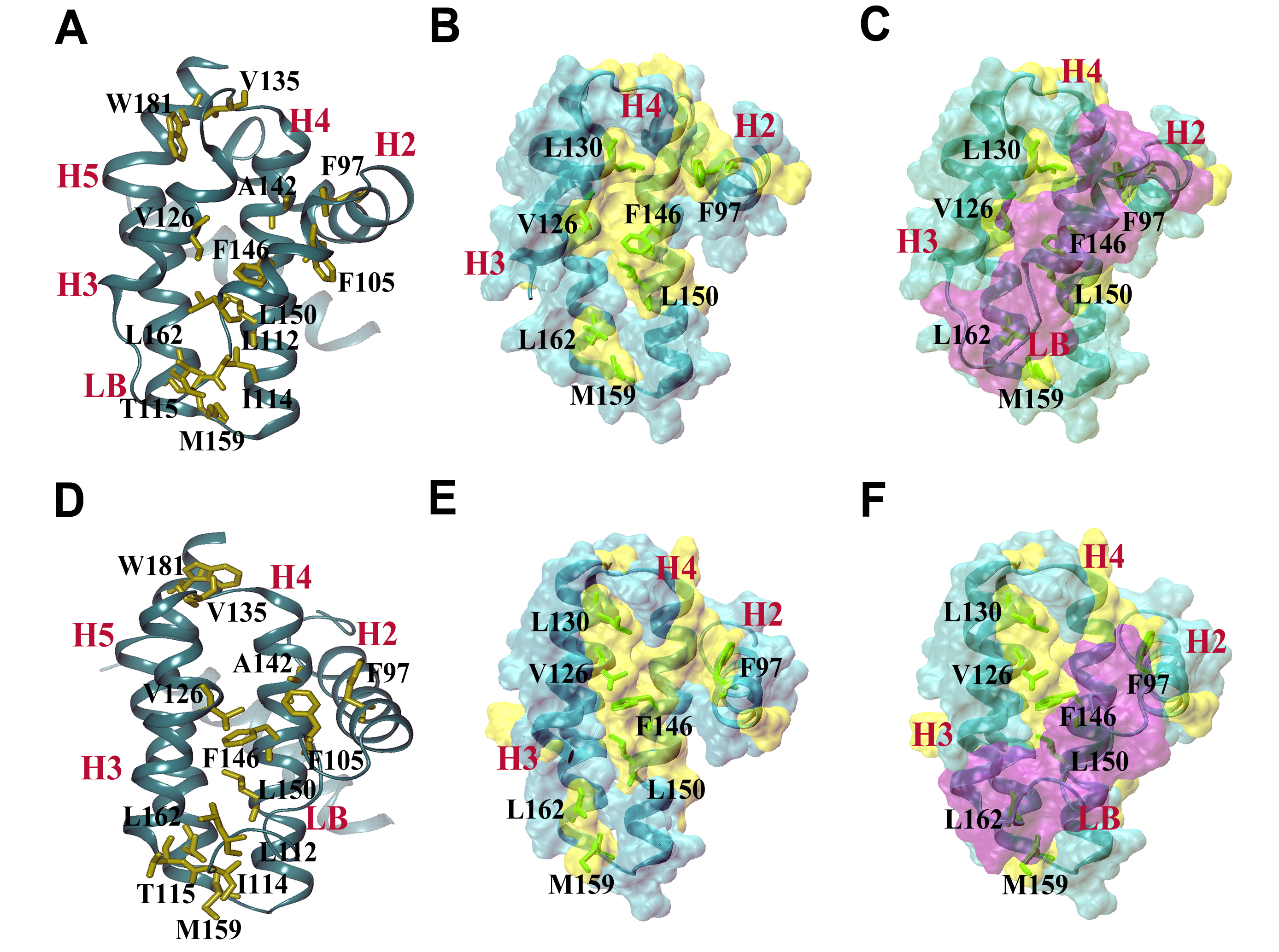

Supplement: Figure S3 — Hydrophobic residues in the hydrophobic cleft: Interactions and accessible surface areas in Apo-pme and Holo-pme-I simulations. Interactions among the hydrophobic residues in the hydrophobic groove are shown for (A) Apo-pme and (B) Holo-pme-I simulations. Helices and side-chains of hydrophobic residues are displayed in ribbon and stick representation respectively. Surface and ribbon representations of helices H2, H3, H4, H5 and loop LD (cyan) along with the hydrophobic residues from these regions (yellow) are shown for (C and E) Apo-pme and (D and F) Holo-pme-I simulations without loop LB (C and D) and with loop LB (E and F). Loop LB surface is represented in purple color in (E) and (F). The Bcl-XL structures shown in this figures were saved at the end of 55 ns production run from Apo-pme and 50 ns production run from Holo-pme-I simulation. (JPG) [file pone.0054397.s003.jpg]

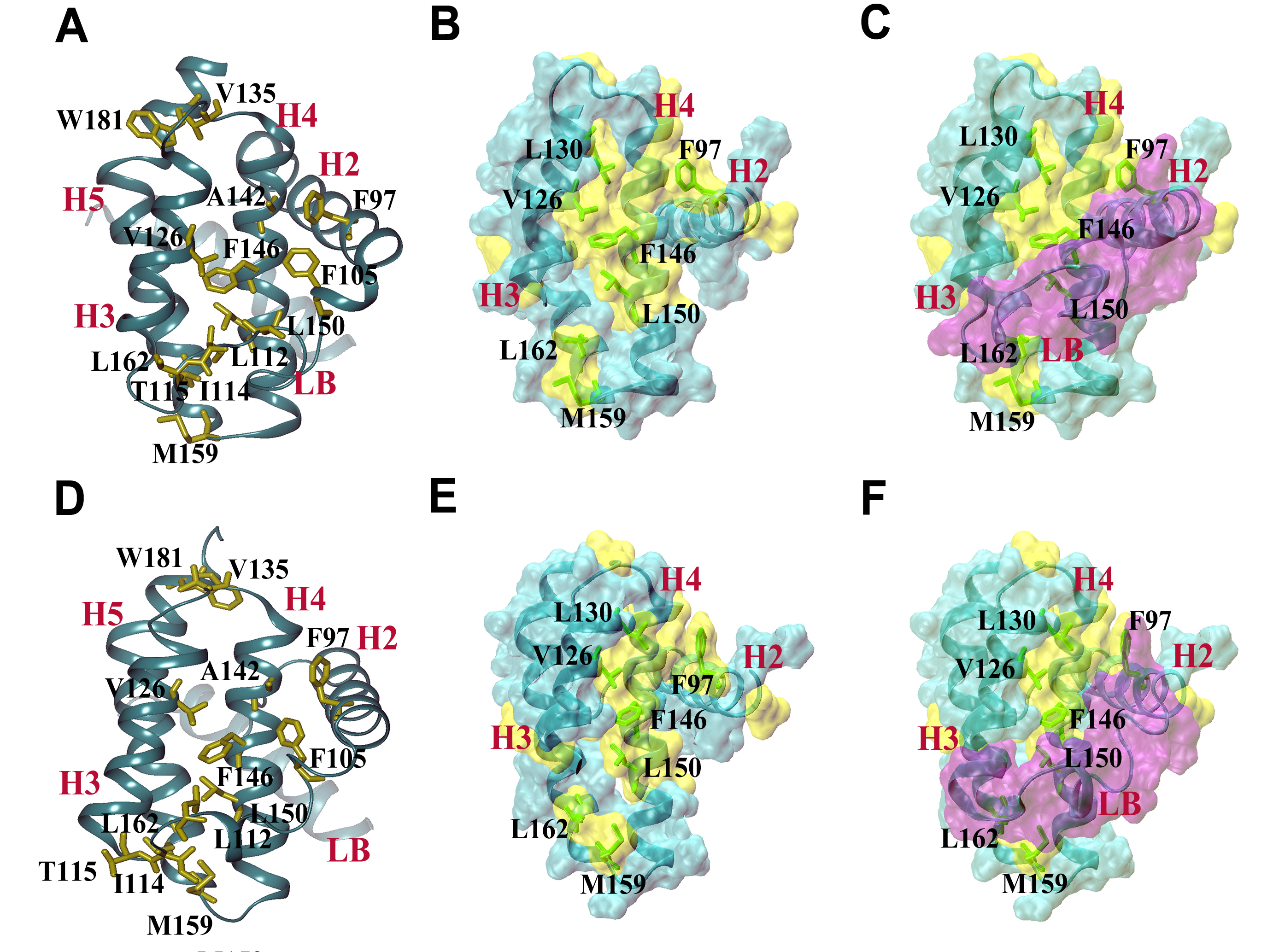

Supplement: Figure S4 — Hydrophobic residues in the hydrophobic cleft: Interactions and accessible surface areas in Holo-pme-II and Holo-pme-III simulations. Interactions among the hydrophobic residues in the hydrophobic groove are shown for (A) Holo-pme-II and (B) Holo-pme-III simulations. Helices and side-chains of hydrophobic residues are displayed in ribbon and stick representation respectively. Surface and ribbon representations of helices H2, H3, H4, H5 and loop LD (cyan) along with the hydrophobic residues from these regions (yellow) are shown for (C and E) Holo-pme-II and (D and F) Holo-pme-III simulations without loop LB (C and D) and with loop LB (E and F). Loop LB surface is represented in purple color in (E) and (F). The Bcl-XL structures shown in this figures were saved at the end of 25 ns production runs from Holo-pme-II and Holo-pme-III simulations. (JPG) [file pone.0054397.s004.jpg]
